# Visual Analysis of Hyperglycemia After Enteral Nutrition in Critically Ill Patients

**DOI:** 10.1155/jnme/5558143

**Published:** 2025-12-23

**Authors:** Rui Zhang, Jinxing Li, Bo Liu, Zhirong Gu, Hou Qiang Huang, Silin Zheng, Min Huang

**Affiliations:** ^1^ School of Nursing, Southwest Medical University, Luzhou, 646000, Sichuan Province, China, swmu.edu.cn; ^2^ Nursing Department, The Affiliated Hospital of Southwest Medical University, Luzhou, 646000, Sichuan Province, China, ahswmu.cn; ^3^ Department of Respiratory and Critical Care Medicine, The Affiliated Hospital of Southwest Medical University, Luzhou, 646000, Sichuan Province, China, ahswmu.cn

**Keywords:** CiteSpace, enteral nutrition, hyperglycemia, intensive care unit (ICU), VOSviewer

## Abstract

**Background:**

Enteral nutrition (EN) is one of the crucial methods in the comprehensive treatment of critically ill patients. However, among critically ill patients receiving EN, hyperglycemia is a common metabolic complication that can lead to adverse clinical outcomes for patients. Therefore, this study aims to provide a comprehensive bibliometric and visual analysis in this field.

**Methods:**

The eligible publications were retrieved from the Web of Science Core Collection (WoSCC) from 2000 to 2023. A bibliometric analysis was performed using CiteSpace and VOSviewer.

**Results:**

A total of 268 articles were analyzed. USA (*n* = 94) had the most contributions in this field. The leading institution was the Royal Adelaide Hospital (*n* = 15) from Australia. The Journal of Parenteral and Enteral Nutrition published the most (*n* = 29). Marianne J Chapman (*n* = 9) was the most frequently published author. Greet Van den Berghe, from Belgium, was the most co‐cited author in this area. According to keyword cluster analysis, diet management is the most widely studied aspect in this field, and EN evaluation is the hotspot and frontier of research.

**Conclusion:**

This is the first bibliometric study to comprehensively summarize the research progress and trend of hyperglycemia after EN in critically ill patients; it provides a valuable reference for researchers interested in this field.

## 1. Introduction

Critical illness patients often experience a hypercatabolic state, accompanied by significantly increased energy and protein consumption, leading to a high prevalence of malnutrition ranging from 38% to 78% [1, 2]. Guidelines recommend initiating enteral nutrition (EN) within 24–48 h after admission, which can significantly improve clinical outcomes [3]. However, hyperglycemia is the most common metabolic complication during EN, with an incidence rate of 21%–30%, and is also an independent risk factor for increased mortality [4]. Currently, studies have primarily focused on intensive insulin therapy, yet most have not fully considered its impact on EN support, often resulting in considerable glycemic variability [4]. How to effectively manage blood glucose while implementing early EN has become a current research focus. Therefore, this study aims to provide a comprehensive analysis of the research status, hotspots, and future trends in this field through bibliometric analysis, thereby offering a reference for further related research.

## 2. Methods and Analysis

### 2.1. Data Sources

The data for this study were sourced from the Web of Science Core Collection (WoSCC) (https://www.webofscience.com/wos/), which is the most widely used database for bibliometric analysis. A studies search was conducted on June 15, 2024, and publications from the period 2000 to 2023 were ultimately included for analysis [5, 6].

### 2.2. Search Strategy

TS=((“intensive care unit^∗^” OR ICU OR “critical care” OR “critically ill” OR “critical illness”)) AND TS=((“enteral nutrition” OR “tube feed^∗^” OR “enteral feed^∗^” OR “nasogastric feed^∗^” OR “gastric feed^∗^”)) AND TS=((“stress hyperglycemi^∗^” OR “stress‐induced hyperglycemi^∗^” OR “stress hyperglycemia” OR “stress hyperglycaemia”)).

### 2.3. Data Extraction

**Figure 1:**
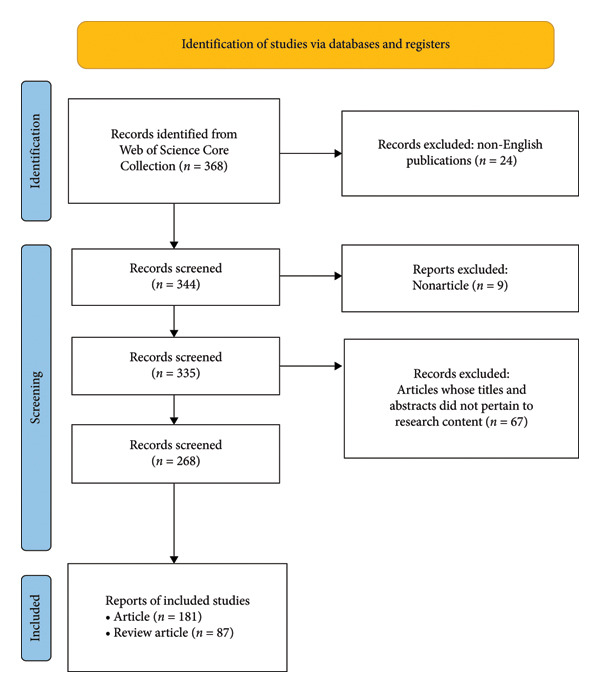
Flowchart of literature search selection.

Two researchers agreed with the search and read the abstracts or full text to classify whether the articles are related to hyperglycemia after EN in critically ill patients. A third investigator resolved any disagreement if a consensus could not be reached. We restricted the publication type to reviews and original articles and set language = English. A flowchart of literature selection is presented in Figure 1.

### 2.4. Data Cleaning

Before initiating the bibliometric analysis, we meticulously processed the raw data using Excel. We classified Taiwan as China, and unified Scotland, Wales, and England into the United Kingdom.

### 2.5. Statistical Analysis

We used Excel for descriptive statistical analysis, including year, journal, citations, and impact factors (***IF***). CiteSpace 6.2.R3 and VOSviewer 1.6.20 were used for bibliometrics and visual analysis. CiteSpace is a Java application for bibliometric analysis developed by Chen [7]. VOSviewer is mainly used for bibliometric network diagram analysis [8]. We used both software programs to analyze countries, institutions, journals, authors, keyword clusters, keyword bursts, and reference bursts.

## 3. Results

### 3.1. The Annual Trend of Articles’ Publication Quantity

**Figure 2:**
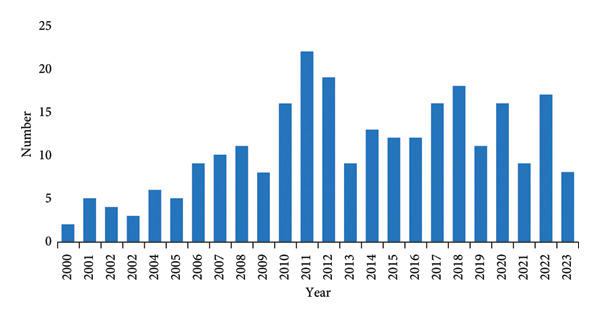
Annual output of hyperglycemia studies after enteral nutrition in critically ill patients.

Based on our search method, 268 articles were published on this topic between 2000 and 2023. Figure 2 indicates that the number of published studies was low between 2000 and 2009, during the early stages, with an average yearly article count of six. Between 2010 and 2023, there was a gradually rising trend in the annual occurrences of hyperglycemia studies after EN in critically ill patients as more researchers began to study this topic.

### 3.2. Countries/Regions’ Network Visualization

**Figure 3:**
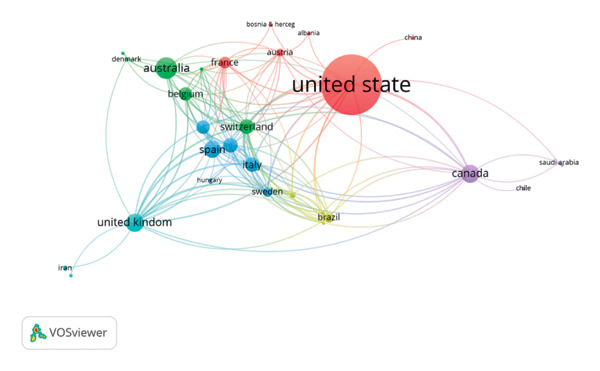
Co‐countries/regions’ network visualization.

We analyzed the literature published in the WoSCC database and utilized the CiteSpace software, selecting countries as nodes. Our analysis includes a total of 38 countries/regions. The top five countries/regions are ranked by the total number of articles published by all authors. Table 1 shows that USA has the most published articles and is the most significant node, with 94 articles (*n* = 4969). Australia and China follow with 26 articles (*n* = 1012) and 23 articles (*n* = 203). A collaborative network was constructed based on the number of articles and relationships in each country, as shown in Figure 3. Through running data analysis, it was found that the international community collaborates in thorough research and cooperation in this specific area. Simultaneously, USA might be seen as the central country in the network, possessing the most prominent nodes and holding the most significant relationships with other countries. Although China has a high ranking in terms of publication quantity, its collaboration with other countries is lacking.

**Table 1:** Top 5 countries according to publication volume.

Rank	Country	Publications	Citations
1	United States	94	4969
2	Australia	26	1012
3	China	23	203
4	United Kingdom	21	2204
5	Canada	21	1971

### 3.3. Visual Analytics for Research Institutions

**Figure 4:**
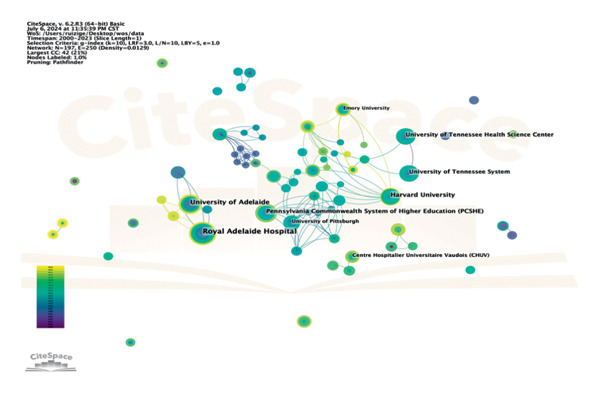
Co‐occurrence of institutions**.**

**Figure 5:**
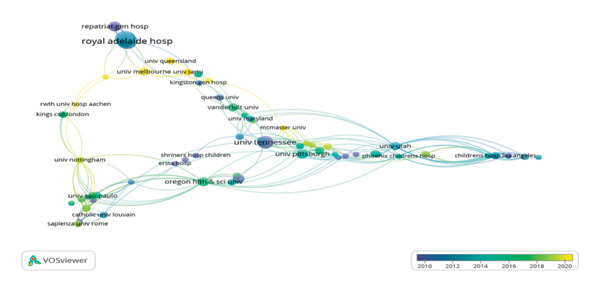
Institutional collaboration timeline.

The network of collaboration and co‐occurrence among study institutions shows their interdisciplinary research capacity and collaboration capacity. 532 institutions in our analysis published articles in this field. The top 5 institutions by number of articles are listed in Table 2. The Royal Adelaide Hospital (*n* = 15) was the institution that published the most articles in this field, followed by the University of Adelaide (*n* = 11), Harvard University (*n* = 10), Pennsylvania Commonwealth System of Higher Education (*n* = 9), and the University of Tennessee (*n* = 8). The most productive institutions mainly come from Australia and USA. Figure 4 illustrates that the most significant connected components of institution co‐occurrence consisted of 197 nodes and 250 links, with a map density of only 0.0129; this low density implies that the quantity of cooperation between these institutions needs to be increased. Figure 5 displays a timeline of collaborations between institutions from 2010 to 2020. The color of the nodes on the map corresponds to the average year in which collaboration occurred for each institution. According to the lower right corner of color gradients, new entries have appeared in universities, including the University of Queensland, the University of Melbourne, and the University of Tartu.

**Table 2:** Top 5 institutions according to publishing volume.

Rank	Institution	Publications	Country
1	Royal Adelaide Hospital	15	Australia
2	University of Adelaide	11	Australia
3	Harvard University	10	United States
4	Pennsylvania Commonwealth System of Higher Education (PCSHE)	9	United States
5	University of Tennessee	8	United States

### 3.4. Analysis of Journals

**Figure 6:**
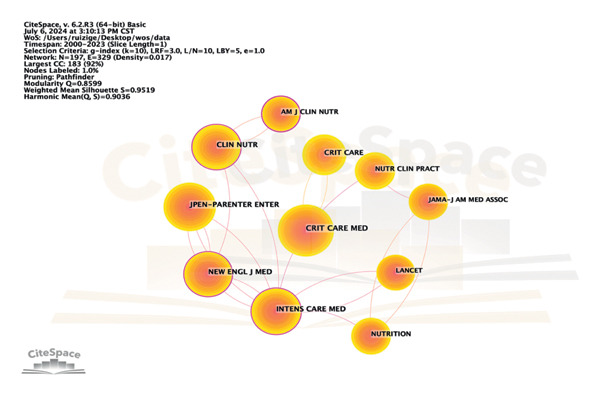
Journal network analysis diagram.

268 articles related to this field have been published in 102 journals. As shown in Table 3, the journal with the greatest number of articles is the Journal of Parenteral and Enteral Nutrition (29 articles, **IF** 2023 = 3.2), followed by Nutrition in Clinical Practice (22 articles, **IF** 2023 = 2.1) and Clinical Nutrition (18 article*s,* **IF** 2023 = 6.6). Among the top five journals, three are Q1 in the Journal Citation Reports (**JCR** Q1∼Q4). A co‐citation relationship exists between two journals when they are cited simultaneously in one or more of the same publications. Figure 6 and Table 4 reveal the source’s co‐citation network. The co‐citation analysis of journals showed that Critical Care Medicine (*n* = 220) was cited the most, followed by the Journal of Parenteral and Enteral Nutrition (*n* = 198) and Intensive Care Medicine (*n* = 173). The top 5 co‐citation journals, except the Journal of Parenteral and Enteral Nutrition, were divided in the Q1 JCR partition.

**Table 3:** Top 5 journals according to publishing volume.

Rank	Journal	Citations	IF (2023)	JCR (2023)
1	Journal of Parenteral and Enteral Nutrition	29	3.2	Q2
2	Nutrition in Clinical Practice	22	2.1	Q3
3	Clinical Nutrition	18	6.6	Q1
4	Critical Care	11	8.8	Q1
5	Critical Care Medicine	10	7.7	Q1

Abbreviations: IF, impact factor; JCR, Journal Citation Reports.

**Table 4:** Top 5 co‐citation journals of publications.

Rank	Journal	Citations	IF (2023)	JCR (2023)
1	Critical Care Medicine	220	7.7	Q1
2	Journal of Parenteral and Enteral Nutrition	198	3.2	Q2
3	Intensive Care Medicine	173	27.1	Q1
4	New England Journal of Medicine	169	96.2	Q1
5	Clinical Nutrition	166	6.6	Q1

Abbreviations: IF, impact factor; JCR, Journal Citation Reports.

### 3.5. Author Distribution

**Figure 7:**
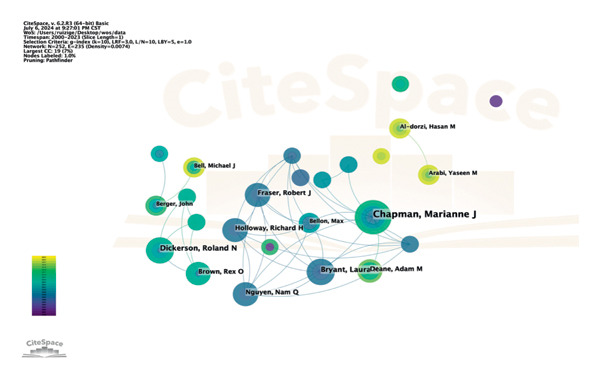
Co‐occurrence of authors.

**Figure 8:**
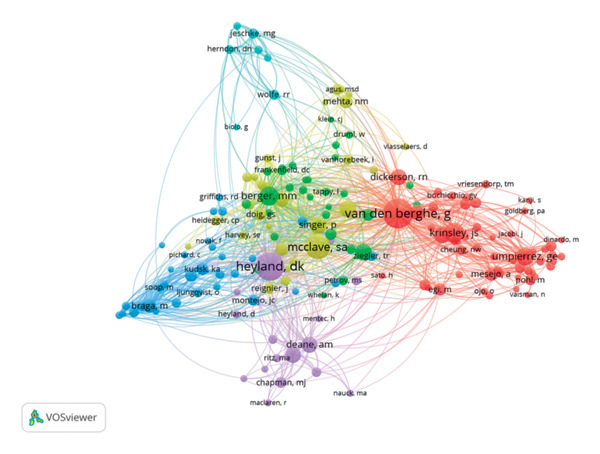
Co‐occurrence of co‐citation authors.

Through our analysis, we found that 252 authors were involved in the study of this field, with a total of 268 articles published. As shown in Figure 7 and Table 5, Marianne J Chapman was the most frequently published author, followed by Roland N Dickerson and Laura K Bryant, who all published 5 articles. The cited authors who appear in the references simultaneously are named co‐cited authors. Among the 6617 co‐cited authors, 3 were co‐cited more than 100 times. We use VOSviewer software to visualize the co‐occurrence of co‐citation authors in this field (Figure 8), in which the top 5 authors cited in total are shown (Table 6). Greet Van den Berghe leads with 197 citations, Daren K Heyland with 180 citations, and Stephen A McClave with 134 citations. Meanwhile, in terms of total link strength (TLS), Greet Van den Berghe (*n* = 3778), Daren K Heyland (*n* = 3196), and Stephen A McClave (*n* = 2388) rank in the top 3 out of the top 5, implying that these authors had closer cooperation with others.

**Table 5:** Top 5 authors according to publishing volume.

Rank	Author	Publications
1	Marianne J Chapman	9
2	Roland N Dickerson	5
3	Laura K Bryant	5
4	Nam Q Nguyen	4
5	Richard H Holloway	4

**Table 6:** Top 5 co‐citation authors of publications.

Rank	Co‐citation author	Country	Citations	TLS
1	Greet Van den Berghe	Belgium	197	3778
2	Daren K Heyland	Canada	180	3196
3	Stephen A McClave	USA	134	2388
4	James S Krinsley	USA	79	2016
5	Guillermo E Umpierrez	USA	86	1553

Abbreviation: TLS, total link strength.

### 3.6. Keyword Cluster

**Figure 9:**
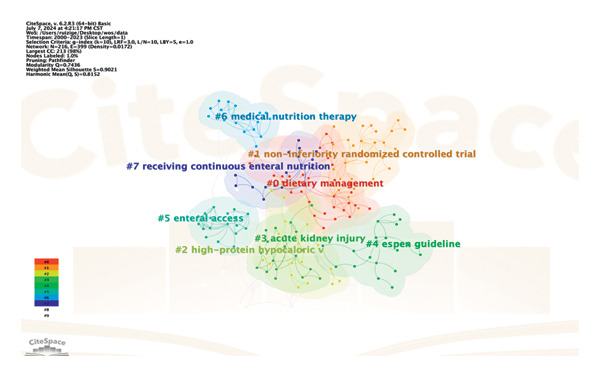
Keyword cluster view.

The CiteSpace software was utilized for keyword clustering analysis on literature related to hyperglycemia following EN in critically ill patients. The keyword cluster aims to identify the main areas of interest and new trends within the field by closely analyzing closely related keywords using measures such as co‐occurrence network density, structure, and centrality. A total of 8 clusters were identified, including dietary management (#0), noninferiority randomized controlled trial (#1), high‐protein hypocaloric, acute kidney injury (#3), The European Society of Clinical Nutrition and Metabolism (#4), enteral access (#5), medical nutrition therapy (#6), and receiving continuous EN (#7). Figure 9 shows numerous connections between nodes, indicating a high degree of co‐occurrence among keywords in this field. The cluster labels are inversely proportional to the size of each cluster, with the largest labeled as dietary management (#0); this suggests that the relationship between dietary management and hyperglycemia after EN in critically ill patients has been thoroughly investigated.

### 3.7. Burst Keywords

**Figure 10:**
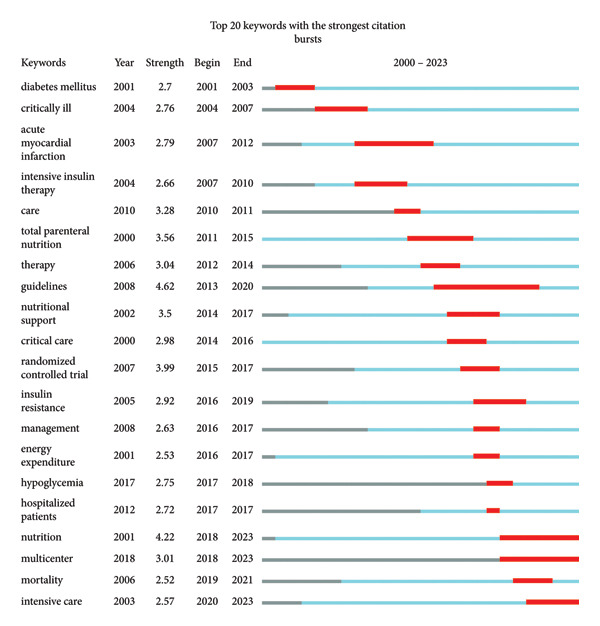
Top 20 keywords with the strongest citation bursts from 2000 to 2023.

Burst keywords are items with rapidly increasing frequency within a short period, and their analysis reflects the evolving trend of research hotspots. They are another important indicator of research frontiers [17, 18]. We utilized CiteSpace software for analysis and identified 20 burst keywords (Figure 10). In our analysis, diabetes was the first keyword to emerge, followed by the significant impact of “guidelines” between 2010 and 2017. In recent years, multicenter research on nutritional treatment of critically ill patients has become a prominent topic in this field.

### 3.8. References Are Co‐Cited for Analysis

**Figure 11:**
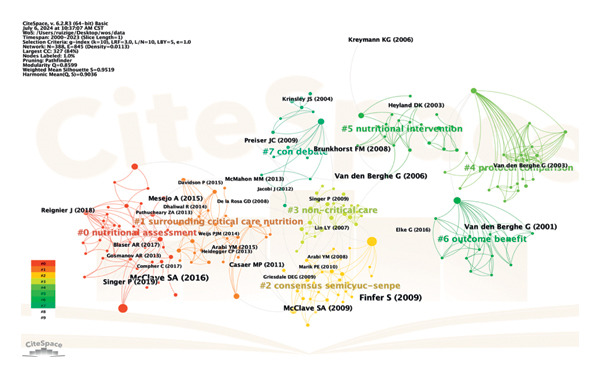
Cluster view of co‐citation of references.

**Figure 12:**
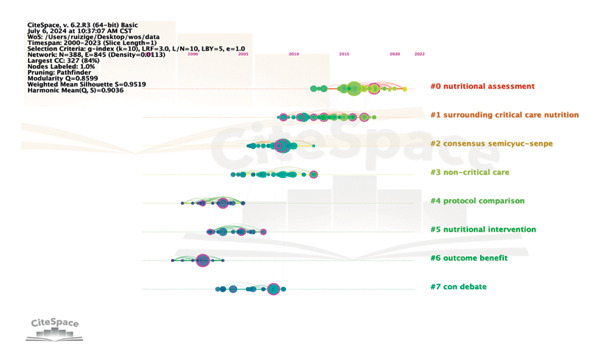
A timeline view of the co‐citation of references.

**Figure 13:**
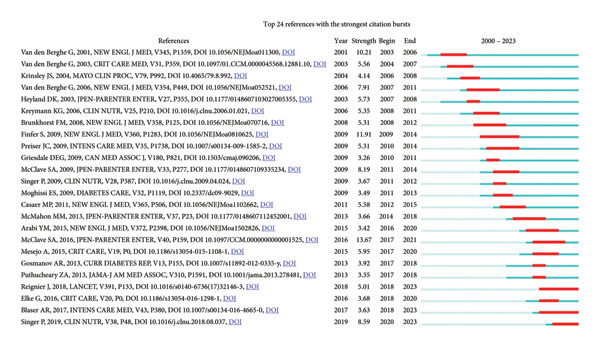
Top 24 references with the strongest citation bursts.

Co‐cited means that two (or more pieces of literature) are cited simultaneously by one or more subsequent literature. In 268 studies on hyperglycemia after EN in critically ill patients, using reference as a node and setting *k* = 10, the generated visual image can obtain 388 nodes and 845 link references in the network (Figure 11). Log‐likelihood ratio (LLR) can be used to cluster the cited literature and generate 8 groups of cluster labels (#0–7), respectively: nutritional assessment (#0), surrounding critical care nutrition (#1), consensus semicyusenpe (#2), noncritical care (#3), protocol comparison (#4), nutritional intervention (#5), outcome benefit (#6), and con debate (#7). The modularity *Q* is 0.8599, which shows that the clustering of the network is reasonable; the silhouette is 0.9519, which indicates that clustering has good homogeneity. Each cluster label is a dominant one by different scholars, and if you are interested in a particular cluster, you can trace its origins. Based on the co‐citation literature, the noun terms in the keywords were extracted, the clustering was annotated, and the timeline diagram of the co‐cited literature was obtained (Figure 12). The distribution from left to right in the center of the node in the figure represents the year in which the cited literature was first published and reflects the temporal characteristics and evolution trend of the cited literature [9]. It can be found that nutritional assessment from 2011 to 2022 is still a research hotspot in this field. Co‐citation is the most important analytical method in bibliometric research, and the frequency of citations can reflect the article’s influence in a specific research field. Using CiteSpace, a burst intensity analysis was conducted on the references, with a time frame set from 2000 to 2023, with a one‐year interval. 24 references with significant burst intensity were identified (Figure 13). The first citation burst occurred in 2001 and was published by Greet Van den Berghe et al. The strongest burst came from an article published in 2016 by Stephen A McClave et al. [9], which published a guideline for providing and evaluating nutritional support in critically ill adults. The three cited literature bursts through 2023 included a multicenter RCT study and two clinical practice guidelines. Three cited literature bursts through 2023 included a multicenter RCT study and two clinical practice guidelines [11, 12].

## 4. Discussion

In this study, we evaluated the current status of the 268 articles by analyzing the field’s institutions, authors, courtiers, and journals. It can help to understand the current information regarding hyperglycemia during EN quickly. It also provides ideas for future research in this area.

Since 2010, annual articles in this field have gradually increased, with USA being the most productive and collaborative country. Among the top contributing institutions, the top two are located in Australia—the Royal Adelaide Hospital and the University of Adelaide. The Journal of Parenteral and Enteral Nutrition published the most articles, while Critical Care Medicine was the most co‐cited journal. Marianne J. Chapman was the most prolific author and Greet Van den Berghe received the most citations.

The incidence of stress hyperglycemia in critically ill patients without a history of diabetes is 30%–47% [13, 14]; poor glycemic control can increase patient morbidity, increase physiological burden, and adversely affect clinical outcomes [15, 16]. Although intensive insulin therapy can effectively lower blood sugar, overly lax management will increase the risk of hypoglycemia, which is also consistent with the keyword clustering [17]. The current guidelines emphasize the importance of safe blood glucose management during EN to avoid excessive fluctuations in blood glucose, which could lead to feeding interruption [18], to ensure the continuity of EN implementation and enable patients to achieve their feeding goals as soon as possible. Studies show that compared with continuous feeding, intermittent feeding can conform to the dietary patterns and digestive functions of the human body, minimize the continuous stimulation of the endocrine system by nutrient solutions, and thereby improve blood sugar control [13, 19]. Meanwhile, when nurses dynamically adjust the feeding rate during intermittent administration, it can also effectively alleviate blood sugar fluctuations. In addition, timely adjusting the type of nutritional preparations based on the patient’s condition, such as special formulas for diabetes, is also helpful for blood sugar control [20]. Therefore, as a group that has closer contact with patients, caregivers play a crucial role in closely monitoring blood sugar levels and communicating with doctors to adjust feeding strategies when necessary.

Some scholars have used machine learning algorithms to predict the insulin demand of critically ill patients during hospitalization [21]. These models aim to predict and evaluate the blood glucose levels of EN in critically ill patients to develop personalized prevention and treatment strategies [22]. This approach could be used to manage this model and incorporate it into clinical decision support systems in multicenter randomized controlled trials at the same time; the management system of hyperglycemia after EN in critically ill patients is yet to be fully standardized. However, studies have shown that care provided by professional teams can shorten hospital stays and improve clinical outcomes such as blood sugar. Multidisciplinary team collaboration (MDT) is a medical model that utilizes various disciplines’ advantages to develop the best personalized plan for patients [23–25]. It is a new clinical approach that complements each other. Therefore, in the future, MDT can be used to manage hyperglycemia accurately after EN in critically ill patients.

## 5. Limitations

This study’s limitation is that only articles from the WoSCC were retrieved, which may lead to incomplete data collection. Meanwhile, software algorithms and the language may also impact the results.

## 6. Conclusions

Nutrition assessment, protein, and blood glucose management are still the main research areas. Meanwhile, understanding the pathological mechanisms of acute stress response can help healthcare professionals establish better EN therapy and improve prognosis. The latest guidelines for critical care nutrition released by the ASPEN in 2022 mention that although many clinical studies have been included, most recommendations still need to be stronger. Therefore, the future aims to explore more large‐scale, high‐quality clinical studies and more suitable new treatment models for managing hyperglycemia after EN in critically ill patients.

## Disclosure

All authors approved the submitted version of this article.

## Conflicts of Interest

The authors declare no conflicts of interest.

## Author Contributions

Rui Zhang planned and conducted this study. Zhirong Gu, Bo Liu, and Jinxing Li gathered the data and drafted the paper. Hou Qiang Huang modified the language and manuscript. Silin Zheng and Min Huang provided guidance. All authors contributed to the submitted version of this article.

## Funding

No funding was received for this manuscript.

## Data Availability

All data generated or analyzed during this study are included in this published article.
